# Development and machine learning-based calibration of low-cost multiparametric stations for the measurement of CO_2_ and CH_4_ in air

**DOI:** 10.1016/j.heliyon.2024.e29772

**Published:** 2024-04-24

**Authors:** R. Biagi, M. Ferrari, S. Venturi, M. Sacco, G. Montegrossi, F. Tassi

**Affiliations:** a Department of Earth Sciences, University of Florence, Via G. La Pira 4, 50121, Firenze, Italy; b Institute of Geosciences and Earth Resources (IGG), National Research Council of Italy (CNR), Via G. La Pira 4, 50121, Firenze, Italy; c Istituto Nazionale di Geofisica e Vulcanologia, Sezione di Palermo, Via Ugo La Malfa 153, Palermo, 90146, Italy; d Department of Physics and Astronomy, University of Florence, Via Sansone 1, 50019, Sesto Fiorentino, Firenze, Italy

**Keywords:** Air quality, Low-cost sensors, Machine learning, Greenhouse gases

## Abstract

The pressing issue of atmospheric pollution has prompted the exploration of affordable methods for measuring and monitoring air contaminants as complementary techniques to standard methods, able to produce high-density data in time and space. The main challenge of this low-cost approach regards the in-field accuracy and reliability of the sensors. This study presents the development of low-cost stations for high-time resolution measurements of CO_2_ and CH_4_ concentrations calibrated via an in-field machine learning-based method. The calibration models were built based on measurements parallelly performed with the low-cost sensors and a CRDS analyzer for CO_2_ and CH_4_ as reference instrument, accounting for air temperature and relative humidity as external variables.

To ensure versatility across locations, diversified datasets were collected, consisting of measurements performed in various environments and seasons. The calibration models, trained with 70 % for modeling, 15 % for validation, and 15 % for testing, demonstrated robustness with CO_2_ and CH_4_ predictions achieving R^2^ values from 0.8781 to 0.9827 and 0.7312 to 0.9410, and mean absolute errors ranging from 3.76 to 1.95 ppm and 0.03 to 0.01 ppm, for CO_2_ and CH_4_, respectively. These promising results pave the way for extending these stations to monitor additional air contaminants, like PM, NO_x_, and CO through the same calibration process, integrating them with remote data transmission modules to facilitate real-time access, control, and processing for end-users.

## Highlights

• Development of low-cost stations for CO_2_ and CH_4_ concentration measurements in air.
• In-field calibrations using a reference instrument in different environmental settings.
• Calibration of sensors based on a machine-learning algorithm.
• ML algorithm providing corrected data for factors that affect the LCSs performances.
• LCSs reliably useful for air quality monitoring.

## Graphical abstract

**Figure:**
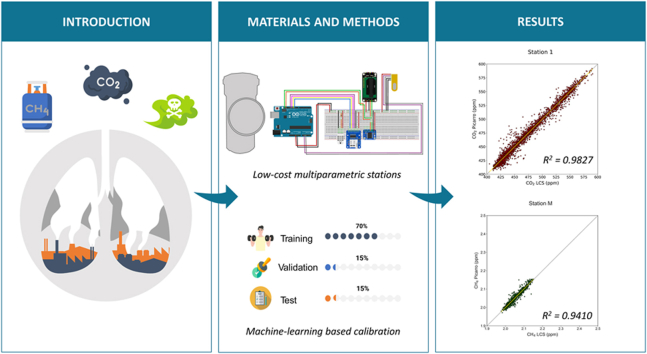

## 1 Introduction

Air quality has emerged as one of the most pressing environmental issues of the modern era, posing significant risks to human health, global climate, and the overall well-being of ecosystems. According to the World Health Organization, almost all of the global population breathes air with levels of harmful pollutants exceeding those recommended by guidelines, causing up to 4.2 million premature deaths worldwide [1] primarily due to the insurgence of cardiovascular and respiratory diseases, e.g. Ref. [2], as well as cancers, e.g., Refs. [3,4]. Furthermore, several drivers of air pollution, e.g., activities involving fossil fuel combustion, contribute significantly to major climate forcers, including carbon dioxide (CO_2_) and methane (CH_4_), which are major greenhouse gases accountable for global warming. Therefore, managing policies to reduce air pollution offers a win-win strategy for climate change mitigation and human health safeguarding. Nevertheless, effective air quality monitoring is crucial. Traditional methods involving sophisticated equipment at stationary monitoring sites, while long-standing, encounter hindrances due to high setup costs and maintenance expenses [5]. This results in insufficient monitoring coverage in rural and non-urban areas, particularly in resource-limited regions and developing countries, leading to limited data resolution in terms of time and space that does not allow to capture the significant variability that atmospheric pollutant concentrations exhibit depending on local sources and features of the surrounding environment [6,7].

In recent years, a paradigm shift in air quality monitoring has occurred with the rise of low-cost sensors (LCSs) for detecting a wide variety of atmospheric pollutants, from particulate matter to gaseous compounds. Gas sensors mostly work on metal oxide semiconductor (MOS) and electrochemical (EC) technologies, whilst non-dispersive infrared (NDIR) and photo-ionization detectors (PID) are other less used technologies. The MOS sensors detect the target gas through the changes in the electrical proprieties (i.e., resistance or conductivity) due to the adsorption of the gas on a semiconductor film exposed to the air [8]. The EC sensors generally operate in amperometry mode, wherein the electrochemical reactions between the target gas and an electrolyte produce a current dependent on the gas concentration [8]. The NDIR technology, widely applied for CO_2_ sensor making [[9], [10], [11]], is based on the spectroscopic principle in which the gas concentrations are proportional to the amount of infrared (IR) light being absorbed by the gas molecules in the air, measured as the difference between the amount of light radiated by the IR lamp and the amount of IR light received by the detector [12,13]. In the PID sensors, the air samples are ionized by UV light; this ionization process leads to the release of electrons and the creation of positively charged ions that generate an electric current signal output. The concentration of the target gas influences the number of ions produced, resulting in a higher or lower current [14].

Extensive research has been conducted on LCSs, e.g. Refs. [5,7,12,[14], [15], [16], [17], [18], [19], [20], [21], [22], [23]], pointing out that the new sensing technologies, though cannot replace traditional equipment, can create new opportunities for broadening access to air quality monitoring. LCSs provide cost-effective means to measure atmospheric pollutant levels in real-time that may enable the tracking of emitting sources [5,24]. This great potential must be accompanied by the evaluation of the accuracy and reliability of data measured by LCSs compared to those of the reference instruments. A shared concern is that these sensors cannot be employed *out-of-the-box* relying on manufacturer-provided conversion models for calibration [25,26], since it cannot be assumed that they exhibit the same responses to the target pollutant under standard conditions and in outdoor environments where they would be applied [27,28]. In fact, LCSs are dependent on environmental temperature and humidity, cross-sensitivity to other species, and their responses can change as they age due to factors like poisoning [5,29]. In the MOS and EC sensors, these limitations are related to the physicochemical properties of the sensors according to the type of electrolyte, electrode, or semiconductor material used, e.g., Refs. [[30], [31], [32]]; whilst the NDIR sensors for CO_2_ undergo cross-sensitivity in presence of high humidity content, since H_2_O absorbs the same infrared wavelength of CO_2_ [12]. Many studies have supported that some of these constraints can be overcome with careful data processing and network design [27,29,[33], [34], [35], [36], [37], [38]]. Given the non-linearity and cross-sensitivity of these sensors, the challenge lies in developing a model that can convert the measured sensor parameter into an output that accounts for external variables. Regression-based models (e.g., linear regression, orthogonal regression, multiple linear regression, polynomial regression) can provide reasonable results and are still widely used for the calibration of LCSs [39]. Despite their many advantages, the calibration coefficients generally change under varying meteorological and microenvironmental conditions, not describing the very complex system of pollutants formation and dispersion in the air [28,40]. Machine learning (ML)-based algorithms have recently emerged as a promising avenue for facing calibration problems, by enhancing the applicability and reducing the effort required in this process [29,41]. Compared to the other methods, ML techniques are problem-specific and data-driven, so usually gain higher accuracies [40]. The general idea of these approaches is to co-locate LCSs next to a reference station and to train a supervised model that can correct the error of the LCSs [18]. There are different categories of supervised learning, the most common are (i) *Random Forest* (RF), an ensemble learning method that works by constructing a multitude of decision trees during the training phase, the results of which are used collectively to produce the final output [42]; (ii) *Gradient Boosted Decision Tree*, as the RF is an ensemble learning method but, instead of combining the different results of multiple decision trees at the end of the process, it combines the results during the process itself [43]; (iii) *Artificial Neural Network*, which are structures consisting of a large number of parallel and strongly interconnected processing units simulating the physiology of the human brain, where each processing unit is similar to a biological neuron and all neurons are organized into layers; the first layer receives input using the activation function and produces outputs, which are analyzed by the next layer of neurons [44].

In this study, we present the assembling of a network of low-cost stations, equipped with NDIR sensors for CO_2_ and MOS sensors for CH_4_, as well as sensors for air temperature and relative humidity, based on Arduino UNO Rev3 microcontroller boards and featured with data loggers. We aim to improve the calibration strategies of low-cost sensors by using the *LinearForestRegressor* (LFR) algorithm, available in the Phyton library *linear-tree* by Cerliani [45], an ensemble machine learning algorithm that combines the strength of Linear Regression Models with the nonparametric learning ability of RF. The choice was driven by the algorithm's relative simplicity and robustness, as well as its rapid data processing time, which are pivotal characteristics for expanding the use of these technologies to (almost) everyone. The calibration approach involved the simultaneous collection of measurements conducted with both the low-cost sensors and the Picarro G2201*i* Cavity Ring-Down Spectroscopy (CRDS) analyzer, used as a reference for CO_2_ and CH_4_, and accounting also for air temperature and relative humidity. In order to develop a general calibration model and mitigate site transferability issues, which refer to the decline in the performance of calibrated devices when moved from one location to another, e.g. Ref. [41], we built the calibration model via a dataset that encompassed several measurements collected in different environments and seasons. This approach broadens the scope of the training dataset to encompass a wide range of concentrations and environmental conditions.

## 2 Materials and methods

### 2.1 Low-cost station design

**Fig. 1:**
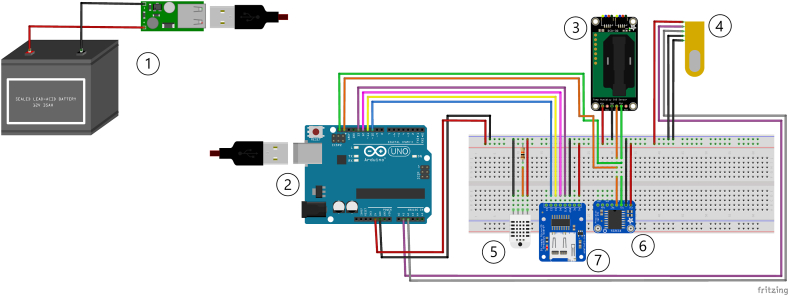
Low-cost stations' design drawn with the open-source software Fritzing (https://fritzing.org/). 1) Power unit; 2) Arduino UNO electronic board; 3) Sensirion SCD30, NDIR sensor for CO_2_ concentrations; 4) Figaro NGM2611-E13 MOS sensor for CH_4_ concentrations; 5) Adafruit DHT22 sensor for air temperature and relative humidity; 6) Adafruit DS3231 Real-Time Clock; 7) Adafruit microSD Break Board data logger. More details are available in the text.

Fig. 1 displays the overall architecture of the low-cost stations. They are powered by a supply unit (1) consisting of a rechargeable 12 V car battery (to be equipped with a solar panel for extended battery life) and a voltage regulator to drop the voltage to 5 V (i.e., the operating voltage of Arduino [46]). Alternatively, they can be powered using a 9 V charging cable to be connected to a 220 V socket, depending on the availability of electrical current. The core of the setup is an Arduino UNO Rev3 board based on the Atmel ATmega328P microcontroller (2) [46]. The board has been programmed through the Arduino IDE software, an integrated development environment in C/C++ (https://www.arduino.cc/en/software), exploiting the manufacturers' libraries and the hosting code available online.

The sensing unit consists of a Sensirion SCD30 sensor for CO_2_ (3), a Figaro NGM2611-E13 sensor for CH_4_ (4), and an Adafruit DHT22 sensor for air temperature (T) and relative humidity (RH) (5). The Sensirion SCD30 is a digital CO_2_ sensor based on NDIR technology. It measures CO_2_ concentrations in the range of 400-10,000 ppm with a declared accuracy of (±30 ppm) and a response time of 2 s [47]. In addition to CO_2_, it measures the temperature (from −40 to 70 °C with an accuracy of ± (0.4 °C + 0.023 × (T [°C] – 25 °C))) and humidity (0–100 % with an accuracy of ±3 % RH) of the surrounding environment using a thermistor and a capacitive humidity sensor, respectively [47]. The sensor communicates via I^2^C or UART bus; in this study, the I^2^C bus connection was used.

The Figaro NGM2611-E13 is an analog module for natural gas alarms based on the Figaro TGS 2611-E00, which operates based on the principle of MOS. Under the presence of CH_4_, the sensing area (a metal oxide semiconductor, such as SnO_2_ or TiO_2_, in the form of granular micro-crystals; see Ref. [15] and references therein for more information) responds to the target gas molecules by exhibiting a proportional decreasing resistance (R_s_) [15,23,48], calculated from the following equation [48]:

$$R_{s}=\left(\frac{V_{c}}{V_{L}}-1\right)\times R_{L}$$

where V_c_ is the total circuit voltage across both the sensing area and the reference resistor (5 V), V_L_ is the output voltage across the reference resistor and varies in response to how the sensing area resistance (R_s_) varies, and R_L_ is a reference resistor connected in series with the sensing area. According to Refs. [15,23], it can be challenging to determine R_L_, so it could be advantageous to calculate the relative sensor response as follows:

$$\frac{R_{S}}{R_{0}}=\frac{\left(\frac{V_{C}}{V_{L}}-1\right)}{\left(\frac{V_{C}}{V_{0}}-1\right)}$$

where R_0_ represents empirical reference resistance corresponding to the lowest measured sensor output voltage in clean air [23]. The R_s_/R_0_ ratio was used to convert sensor signal output to CH_4_ concentration readings in the calibration procedure (Section 2.2). The sensor underwent a factory calibration at 5,000 ppm, 20 °C, and 65 % of RH, and the detection range specified by the manufacturer is 500–10,000 ppm [48]. While this mole fraction is not relevant for atmospheric concentration applications, the NGM2611-E13 was successfully used for measuring indoor [49] and outdoor [15] ambient concentrations of methane (2–9 ppm), and for flux measurements from water bodies [23,50].

The Adafruit DHT22 employs a capacitive humidity sensor and a thermistor for the measurement of ambient conditions. It measures relative humidity within a range of 0–100 % and an accuracy of 2–5%, and temperature spanning from −40 to 80 °C with an accuracy of ±0.5 °C. The sensor then generates a digital signal on the data pin [51].

The architecture includes also an Adafruit DS3231 Real-Time Clock (RTC) as a precise temporal reference (6). Finally, the data logger, consisting of an Adafruit MicroSD Breakout Board (7), allows the recording and storing of data collected by the sensors with a time resolution of 10 s in a text file on a micro-SD card. A technical note with the circuit scheme and the programming code is accessible in the Supplementary Material.

### 2.2 Calibration procedure

#### 2.2.1 Measuring instruments

Aiming to correct the response of the low-cost stations in real-world environments, we developed a calibration procedure based on a ML algorithm using data measured by six low-cost stations for CO_2_ and CH_4_ (hereafter named stations M (*mother station*), 1, 2, 3, 4, and 5; the architecture and functions are explained in Section 2.1) and a Picarro G2201*i*, the latter being used as a reference instrument. The Picarro G2201*i* (hereafter referred to as Picarro) is a high-frequency (1 measure per second) CRDS analyzer of CO_2_ and CH_4_ concentrations (in ppm). Its operating interval ranges from 380 (average atmospheric values) to 2,000 ppm for CO_2_, from 1.8 to 12 ppm for CH_4_ in high-precision mode, and from 10 to 1,000 ppm for CH_4_ in high-range mode [52]. The Picarro's calibration was performed at the beginning of each measuring period using the following standards (Air Liquide): (i) 380, 500, and 1,000 ppm CO_2_, (ii) 1.8, 5, and 10 ppm CH_4_. The precision was within 0.2 ppm CO_2_ and 0.05 ppm CH_4_. The instrument was further checked at the end of the measurements.

#### 2.2.2 Measuring sites

One of the major concerns when calibrating LCSs regards site transferability, i.e., moving a calibrated device from the location where the calibration has been performed to another one, which usually leads to a performance loss due to measurement conditions beyond the training domain [41], and reference therein. To mitigate this issue and create cost-effective stations capable of delivering robust performance in different locations, i.e. encompassing a wide range of concentrations and ambient conditions, the datasets used for ML-based calibration procedure included measurements performed in different seasons and a variety of environmental settings, as follows: (i) Municipality of Scandicci (Metropolitan area of Florence, Tuscany), representing a widely urbanized and industrialized area; (ii) locality of Galluzzo (south of Florence, Tuscany), chosen as a sub-urban site; (iii) localities of Renazzo and (iv) Barbiano in the Po Plain (the first in Ferrara Province, and the latter located in Ravenna Province, Emilia-Romagna), characterized by the presence of two domestic wells emitting notably high concentrations of CH_4_ (up to 16 ppm of CH_4_ measured in air); (v) Vulcano Island (Aeolian Archipelago, Sicily) and (vi) Municipality of Pozzuoli (Naples, Campania), chosen as hydrothermal end-members characterized by considerable concentrations of H_2_S in the air (up to hundreds of ppb), which can possibly act as an interference species for LCS; (vii) an industrial plant extracting and refining CO_2_ in the Municipality of Montepulciano (Siena, Tuscany); (viii) the Padule di Fucecchio wetland, the largest Italian inner wetland, stretching between the provinces of Florence, Pistoia, Lucca and Pisa (northwestern Tuscany). It should be pointed out that, due to sporadic malfunctions in some stations, the calibration datasets varied in the amount of data, both for CO_2_ and CH_4_. The measurements covered the summer, fall, and winter of 2022, and the winter and part of spring of 2023. Minute-averages were obtained from the datasets acquired from each sampling site, both for the low-cost stations and the reference instrument, and further used for the calibration treatment.

#### 2.2.3 Calibration methods

**Fig. 2:**
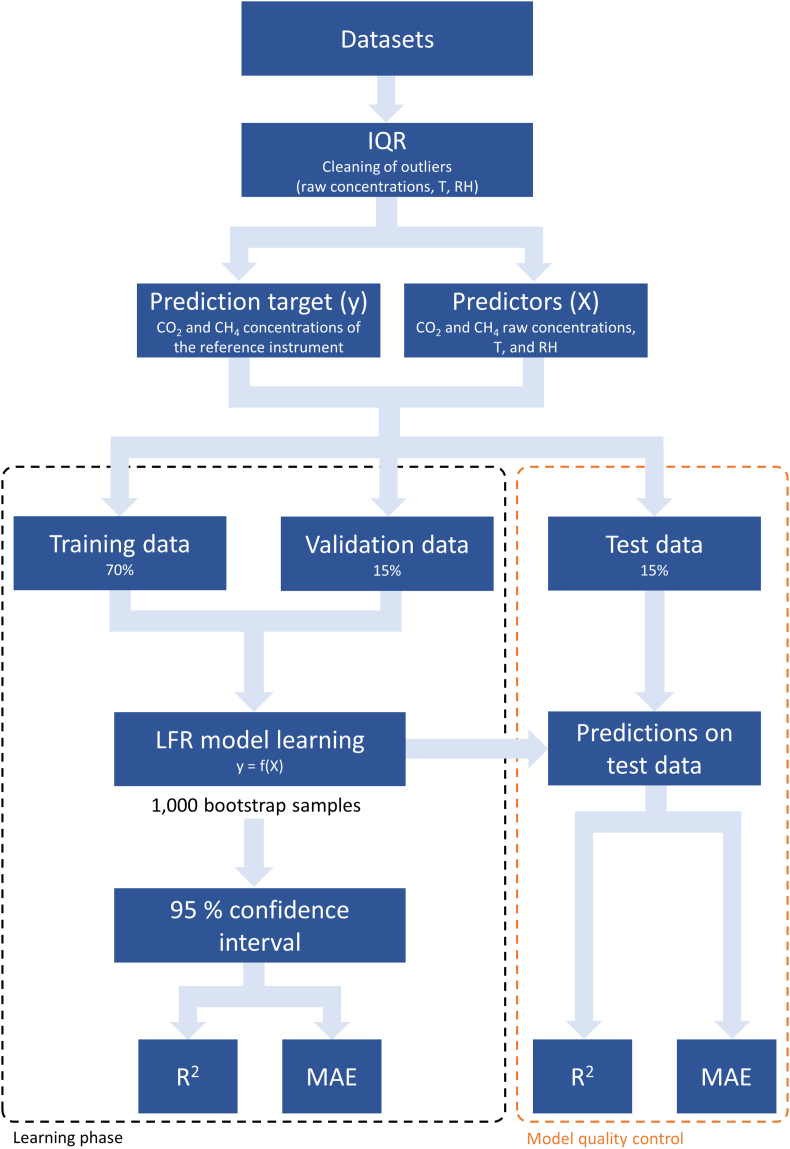
Scheme of the calibration procedure. Six low-cost stations for CO_2_ and CH_4_ were co-located with a reference measurement station (Picarro G2201*i*). Air temperature and relative humidity were also measured as key environmental variables that can disturb the sensors' signal outputs. The low-cost sensor signal (i.e., CO_2_ concentrations and R_s_/R_0_ ratio for CO_2_ and CH_4_ sensors, respectively), which have been generically renamed *raw concentrations*, and the environmental variables were set as predictors (X), or features of the models, whilst the reference station signal represented the prediction target (y). The time resolution was set to minute averages. We trained separate calibration datasets for each CO_2_ and CH_4_ sensor with the Linear Forest Regression (LFR) machine learning algorithm. The training models were evaluated using the R^2^ coefficient and the mean absolute error (MAE), assessing the 95 % confidence interval through the bootstrap technique (1,000 bootstrap samples). The ability of the model to predict unknown data was evaluated on out-of-sample test data, i.e. on data that were not used during the training phases, using the R^2^ coefficient and the MAE.

The calibration models were constructed using the LFR algorithm, available in the library *linear-tree* for Phyton by Cerliani (2022) (https://github.com/cerlymarco/linear-tree). The LFR is an ensemble machine learning algorithm, revised starting from the work of [53], which generalizes the well-known RF algorithm by combining it with linear models. RF is one of the best-performing learning algorithms in environmental science since it easily adapts to nonlinearities found in environmental data [54]. It is a supervised algorithm based on the construction of multiple decision trees that follows the concept of ensemble learning, where the combination of multiple ML models results in predictions that are more reliable than those of individual models. Each decision tree consists of a series of nodes, which branch out into multiple tree levels until reaching the final one, known as the leaf node. In each leaf node, there are at least one or more samples extracted from the training data. The prediction made by each tree for any set of predictors is determined by calculating the average of these samples [41]. To prevent the trees from becoming correlated with each other, RF enhances their diversity by having them grow from distinct training data subsets. This is achieved through a process known as bagging, which involves the creation of training data by repeatedly sampling from the original dataset with replacement. In other words, data is drawn from the initial sample to form the next subset, with no data being permanently removed from the input sample. Consequently, some data may be included multiple times during training, while others may not be used at all. Thus, greater stability is achieved [55]. However, being a completely non-parametric predictive algorithm, RF may display some limitations in describing the relationship between the response and the predictors, running into issues of underfitting, which occurs when the model is too simple to capture the complexity of the data, or overfitting, i.e. when the model is too complex and fits the training data too closely, but generalizes poorly to new data. Moreover, RF is not able to perform extrapolation when predictions are required on data that fall outside the domain of the training dataset. To address these limitations and achieve an accurate model over a wider concentration range, the LFR algorithm first fits a linear model on the whole dataset, then a RF is trained on the same dataset but using the residuals of the previous steps as the target. The final predictions are the sum of the raw linear predictions and the residuals modeled by the RF [45]. In this way, the strength of linear models improves the nonparametric learning ability of tree-based algorithms. The signals from the low-cost sensors (i.e., CO_2_ concentrations and R_s_/R_0_ ratio for CO_2_ and CH_4_ sensors, respectively), which have been generically renamed *raw concentrations* in Fig. 2, and the environmental variables were set as predictors (X), or features of the models, whilst the reference station signal represented the prediction target (y).

Before the construction of the predictive models, the entire datasets were processed to clean from outliers through the interquartile range statistical method (IQR). Then, datasets were divided into three parts to construct the predictive models: training, validation, and test data. The training and validation datasets were used during the learning phase. The test dataset was used afterward to evaluate the quality of the model. In this way, it was possible to determine the ability of the model to predict new cases not used during the learning phase. The training datasets were 70 % of the primary datasets, whereas the test and validate datasets included the remaining 15 % and 15 %, respectively. This type of splitting is commonly used in the supervised training of ML models [[56], [57], [58]], allowing sufficient data for training and model quality control. The degrees of freedom of the algorithms were tuned by selecting the best hyperparameter values through the *GridSearchCV* function, (available in the *Scikit-Learn* library for Phyton). These hyperparameters control the growth of the random forest and the shape of decision trees, avoiding the overfitting problem and obtaining a model with good generalization capability, i.e., the ability to transfer the high accuracy achieved in the training phase to the test one. The training models and test data were evaluated through the coefficient of correlation R^2^ and the mean absolute error (MAE). To assess the 95 % confidence interval of R^2^ and MAE in the training datasets, bootstrap elaborations were performed with the construction of 1,000 samples through resampling. This process involved repeatedly selecting and training the model on different subsets of the training data, allowing us to capture a range of performance outcomes and quantify the uncertainty associated with our R^2^ and MAE estimates. A scheme of the procedure's steps is reported in Fig. 2.

## 3 Results

In the following sections, the summary descriptive statistical parameters of the calibration datasets are reported for each station (Table 1 and Table 2). Counts, minimum, maximum, mean, and standard deviation values of CO_2_ and CH_4_ concentrations measured by the low-cost stations (referred to as *CO*_*2*_*_station* and *R*_*s*_*/R*_*0*_, respectively in Table 1 and Table 2, and hereafter broadly referred in the text to as *raw concentrations*), as well as those relative to the reference instrument (*CO*_*2*_*_Picarro*, Table 1, and *CH*_*4*_*_Picarro*, Table 2), are described together with the environmental parameters (T and RH).

**Table 1:** Summary descriptive statistical parameters of CO_2_ datasets collected for each station and used for the calibration procedure. The concentrations of CO_2_, of both the Picarro reference instrument and the low-cost stations, are in ppm; temperature is in °C; relative humidity is in %.

**Station 1**					**Station 2**				
	**CO₂_Picarro**	**CO₂_station**	**T**	**RH**		**CO₂_Picarro**	**CO₂_station**	**T**	**RH**
**units**	(ppm)	(ppm)	(°C)	(%)	**units**	(ppm)	(ppm)	(°C)	(%)
**count**	30377	30377	30377	30377	**count**	10732	10732	10732	10732
**mean**	467	518	22.2	60	**mean**	433	485	31.5	38
**std**	43.6	49.0	5.5	15.9	**std**	14.5	25.0	5.2	13.6
**min**	404	397	7.1	14	**min**	409	406	13.4	14
**25 %**	430	479	18.4	48	**25 %**	422	467	27.6	26
**50 %**	457	510	20.8	67	**50 %**	428	479	31.7	36
**75 %**	495	554	25.6	72	**75 %**	437	505	35.6	47
**max**	592	654	37.8	79	**max**	475	558	47.2	90
**Station 3**					**Station 4**				
	**CO₂_Picarro**	**CO₂_station**	**T**	**RH**		**CO₂_Picarro**	**CO₂_station**	**T**	**RH**
**units**	(ppm)	(ppm)	(°C)	(%)	**units**	(ppm)	(ppm)	(°C)	(%)
**count**	9340	9340	9340	9340	**count**	9459	9459	9459	9459
**mean**	430	628	32.2	35	**mean**	429	337	31.8	36
**std**	10.6	43.6	5.0	11.9	**std**	10.1	20.0	5.0	12.7
**min**	406	517	16.2	13	**min**	409	276	21.2	14
**25 %**	422	592	28.3	24	**25 %**	422	323	28.1	25
**50 %**	426	621	32.6	33	**50 %**	426	333	32.4	34
**75 %**	433	665	36.2	42	**75 %**	433	349	35.7	46
**max**	460	778	43.4	73	**max**	459	406	41.8	78
**Station 5**					**Station M**				

	**CO₂_Picarro**	**CO₂_station**	**T**	**RH**		**CO₂_Picarro**	**CO₂_station**	**T**	**RH**
**units**	(ppm)	(ppm)	(°C)	(%)	**units**	(ppm)	(ppm)	(°C)	(%)
**count**	9398	9398	9398	9398	**count**	9765	9765	9765	9765
**mean**	429	506	31.6	36	**mean**	430	362	30.7	41
**std**	9.4	21.0	5.2	12.4	**std**	10.8	16.7	5.2	13.2
**min**	407	445	16.5	14	**min**	407	314	14.1	15
**25 %**	422	492	27.8	25	**25 %**	422	350	26.7	29
**50 %**	426	504	32.5	34	**50 %**	426	360	31.3	39
**75 %**	432	520	35.7	45	**75 %**	433	373	34.5	50
**max**	457	575	42.0	77	**max**	462	419	42.1	77

**Table 2:** Summary descriptive statistical parameters of CH_4_ datasets collected for each station and used for the calibration procedure. R_s_/R_0_ is the relative sensor response (see Section 2.1) and is a pure number. The concentrations of CH_4_, of both the Picarro reference instrument and the low-cost stations, are in ppm; temperature is in °C; and relative humidity is in %.

**Station 1**					**Station 2**				
	**CH₄_Picarro**	**Rₛ/R₀**	**T**	**RH**		**CH₄_Picarro**	**Rₛ/R₀**	**T**	**RH**
**units**	(ppm)		(°C)	(%)	**units**	(ppm)		(°C)	(%)
**Count**	29607	29607	29607	29607	**count**	3221	3221	3221	3221
**mean**	2.14	0.78	22.0	61	**mean**	2.05	0.46	33.2	40
**std**	0.096	0.064	5.3	15.8	**std**	0.057	0.026	4.2	11.7
**min**	1.99	0.595	6.7	14	**min**	1.96	0.40	25.8	19
**25 %**	2.05	0.733	18.4	50	**25 %**	2.00	0.44	29.7	30
**50 %**	2.12	0.764	20.6	67	**50 %**	2.04	0.46	33.0	41
**75 %**	2.21	0.817	25.2	72	**75 %**	2.09	0.49	37.0	49
**max**	2.42	0.944	38.5	79	**max**	2.23	0.54	42.4	62
**Station 3**					**Station 4**				
	**CH₄_Picarro**	**Rₛ/R₀**	**T**	**RH**		**CH₄_Picarro**	**Rₛ/R₀**	**T**	**RH**
**units**	(ppm)		(°C)	(%)	**units**	(ppm)		(°C)	(%)
**count**	3228	3228	3228	3228	**count**	3230	3230	3230	3230
**mean**	2.05	0.87	33.5	40	**mean**	2.05	0.34	33.1	41
**std**	0.057	0.05	4.1	11.4	**std**	0.057	0.020	4.2	11.7
**min**	1.96	0.72	26.3	18	**min**	1.96	0.28	25.4	20
**25 %**	2.00	0.83	30.1	30	**25 %**	2.00	0.33	29.4	31
**50 %**	2.04	0.85	33.3	40	**50 %**	2.04	0.34	33.3	41
**75 %**	2.09	0.92	36.9	49	**75 %**	2.09	0.36	36.8	49
**max**	2.22	1	43.4	60	**max**	2.23	0.39	41.8	62
**Station 5**					**Station M**				
	**CH₄_Picarro**	**Rₛ/R₀**	**T**	**RH**		**CH₄_Picarro**	**Rₛ/R₀**	**T**	**RH**
**units**	(ppm)		(°C)	(%)	**units**	(ppm)		(°C)	(%)
**count**	10337	10337	10337	10337	**count**	9810	9810	9810	9810
**mean**	2.04	0.35	31.2	37	**mean**	2.05	0.07	30.4	41
**std**	0.04	0.024	5.1	13.1	**std**	0.038	0.004	5.2	13.9
**min**	1.96	0.26	20.6	14	**min**	1.97	0.06	20.5	15
**25 %**	2.01	0.33	27.2	26	**25 %**	2.01	0.07	26.3	29
**50 %**	2.03	0.35	31.7	37	**50 %**	2.03	0.07	30.4	41
**75 %**	2.07	0.37	35.4	46	**75 %**	2.07	0.07	34.5	51
**max**	2.15	0.41	42.0	77	**max**	2.15	0.08	42.1	77

### 3.1 CO_2_ and environmental parameters datasets

The dataset gathered for station 1 was the broadest one, with a total of 30,377 data, and presented the widest CO_2_ concentration range measured by the Picarro (varying from 409 to 475 ppm, mean value: 467 ppm, standard deviation: 43.6 ppm), whilst the raw concentrations, recorded by the CO_2_ low-cost sensor, ranged from 404 to 592 ppm, with a mean value of 518 ppm and a standard deviation of 49.0 ppm. Temperature and relative humidity ranged from 7.1 to 37.8 °C, and from 14 to 79 %, respectively (mean values of 22.2 °C and 60 %, and standard deviations of 5.5 °C and 15.9 %, respectively) (Table 1).

The dataset collected for station 2 (10,732 data) displayed CO_2_ concentrations from 409 to 475 ppm (mean value: 433 ppm, standard deviation: 14.5 ppm), and from 406 to 558 ppm (mean value: 485, standard deviation: 25.0), for the Picarro and the low-cost sensor, respectively. The temperature reached a minimum value of 13.4 °C and a maximum of 47.2 °C (mean value: 31.5 °C, standard deviation: 5.2 °C), while the relative humidity ranged from 14 to 90 % (mean value: 38 %, standard deviation: 13.6 %) (Table 1).

Concerning station 3, the model was built on a dataset of 9,340 data, with CO_2_ concentrations between 406 and 460 ppm for the Picarro (mean value: 430 ppm, standard deviation: 10.6 ppm), and raw concentrations between 517 and 778 ppm (mean value: 628 ppm, standard deviation: 43.6 ppm). The temperature and relative humidity ranged from 16.2 to 43.4 °C (mean value: 32.2 °C, standard deviation: 5.0 °C) and from 13 to 73 % (mean value: 35 %, standard deviation: 11.9 %), respectively (Table 1).

Station 4's dataset (9,459 counts) displayed CO_2_ concentrations measured by the Picarro ranging from 409 to 459 ppm, with a mean value of 429 ppm and a standard deviation of 10.1 ppm, and CO_2_ raw concentrations ranging from 276 to 406 ppm, with a mean value of 337 ppm and a standard deviation of 20.0 ppm. The temperature varied from a minimum of 21.2 °C to a maximum of 41.8 °C, with a mean value of 31.8 °C (standard deviation: 5.0 °C). The relative humidity ranged from 14 to 78 %, with a mean value of 36 % and a standard deviation of 12.7 % (Table 1).

The dataset of Station 5 included 9,398 data. CO_2_ concentrations of Picarro ranged from 407 to 457 ppm (mean value: 429 ppm, standard deviation: 9.4 ppm), whilst those of the low-cost sensor were from 445 to 575 ppm, with a mean value of 506 ppm and a standard deviation of 21.0 ppm. The temperature and relative humidity varied from 16.5 to 42 °C, and from 14 to 77 %, respectively, with mean values of 31.6 °C (standard deviation: 5.2 °C) and 36 % (standard deviation: 12.4 %), respectively (Table 1).

Finally, station M's dataset was made of 9,765 data, displaying CO_2_ concentrations that varied from 407 to 462 ppm for the Picarro (mean value: 430 ppm, and standard deviation: 10.8 ppm), and from 314 to 419 ppm for the low-cost sensor (mean value: 362 ppm, and standard deviation: 16.7 ppm). The temperature was on average 30.7 °C (standard deviation: 5.2 °C), ranging between 14.1 and 42.1 °C; whilst the relative humidity ranged from 15 to 77 %, with a mean value of 41 % and a standard deviation of 13.2 % (Table 1).

### 3.2 CH_4_ and environmental parameters datasets

Analogously to CO_2_, different datasets specific to each station were gathered for CH_4_ concentrations, raw data, and the relative environmental parameters to train and evaluate the calibration models, wherein the R_s_/R_0_ ratios were used to convert the sensor signal output (see Section 2.1) to CH_4_ concentrations.

As it was for CO_2_, the dataset for CH_4_ calibration relative to station 1 was the largest, including 29,607 data, with CH_4_ concentration values measured by the Picarro ranging from 1.99 to 2.42 ppm (mean value: 2.14 ppm, standard deviation: 0.096 ppm), and raw values (R_s_/R_0_) measured by the low-cost sensor ranging from 0.59 to 0.94 (mean value: 0.78, standard deviation: 0.064). The environmental parameters varied from 6.7 to 38.5 °C (mean value: 22.0 °C, standard deviation: 5.3 °C) for the temperature, and from 14 to 79 % (mean value: 61 %, standard deviation: 15.8 %) for the relative humidity (Table 2).

Station 2's dataset, counting 3,221 data, displayed CH_4_ concentrations between 1.96 and 2.23 ppm (mean value: 2.05 ppm, standard deviation: 0.057 ppm), and R_s_/R_0_ values ranging from 0.40 to 0.54 (mean value: 0.46, standard deviation: 0.026). The temperature was on average 33.2 °C, with a minimum of 25.8 °C and a maximum of 42.4 °C (standard deviation: 4.2 °C), while relative humidity varied from 19 to 62 %, with a mean value of 40 % and a standard deviation of 11.7 % (Table 2).

For station 3, the calibration dataset consisted of 3,228 data, with Picarro's CH_4_ concentrations ranging from 1.96 to 2.22 ppm (mean value: 2.05 ppm, standard deviation: 0.057 ppm), and R_s_/R_0_ ratios from 0.72 to 1 (mean value: 0.87, standard deviation: 0.05). The temperature varied between 26.3 and 43.4 °C, with a mean value of 33.5 °C (standard deviation: 4.1 °C), whilst the relative humidity ranged from 18 to 60 %, with a mean value of 40 % (standard deviation: 11.4 %) (Table 2).

Station 4 (3,230 data) displayed mean values of 2.05 ppm for CH_4_ concentrations recorded by the Picarro (minimum value: 1.96 ppm, maximum value: 2.23 ppm, standard deviation: 0.057 ppm), and 0.34 for the R_s_/R_0_ ratio (minimum value: 0.28, maximum value: 0.39, standard deviation: 0.020). Temperature and relative humidity of air varied from 25.4 to 41.8 °C, and from 20 to 62 %, respectively, with means values of 33.1 °C (standard deviation: 4.2 °C) and 41 % (standard deviation: 11.7 %), respectively (Table 2).

In the calibration dataset collected for station 5 (counting 10,337 data), CH_4_ concentrations varied from 1.96 to 2.15 ppm, with a mean value of 2.04 ppm and a standard deviation of 0.04 ppm. Meanwhile, the raw data of the low-cost sensor ranged from 0.26 to 0.41, with a mean value of 0.35 and a standard deviation of 0.024. The mean value of the temperature was 31.2 °C, with values ranging from 20.6 to 42.0 °C (standard deviation: 5.1 °C), while relative humidity was between 14 and 77 %, with a mean value of 37 % and a standard deviation of 13.1 % (Table 2).

Finally, for Station M a total of 9,810 data were gathered for the calibration dataset, with CH_4_ concentrations acquired by the Picarro ranging from 1.97 to 2.15 ppm (mean value: 2.05 ppm, standard deviation: 0.038 ppm), and the R_s_/R_0_ ratio from 0.06 to 0.08 (mean value: 0.07, standard deviation: 0.004). The environmental parameters varied from 20.5 to 42.1 °C for the temperature and from 15 to 77 % for the relative humidity, with mean values of 30.4 °C (standard deviation: 5.2 °C) and 41 % (standard deviation: 13.9 %), respectively (Table 2).

## 4 Discussion

### 4.1 Assessing models fit on training data

**Fig. 3:**
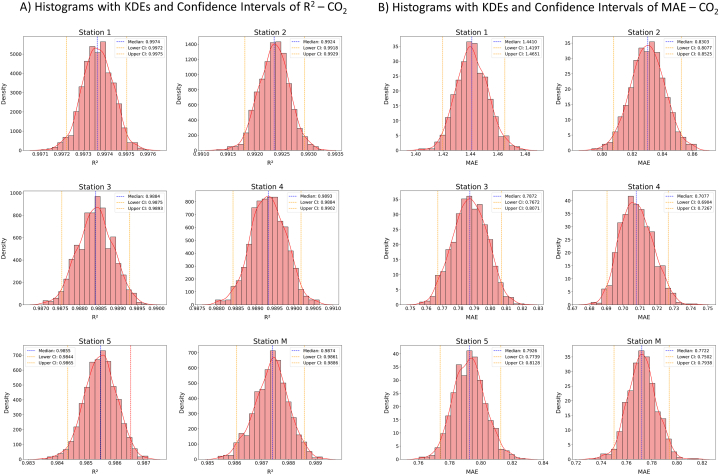
A) R^2^ across 1,000 bootstrap samples of the training models for CO_2_, showing median and 95 % confidence intervals. B) MAE across 1,000 bootstrap samples of the training models for CO_2_, showing median and 95 % confidence intervals.

**Fig. 4:**
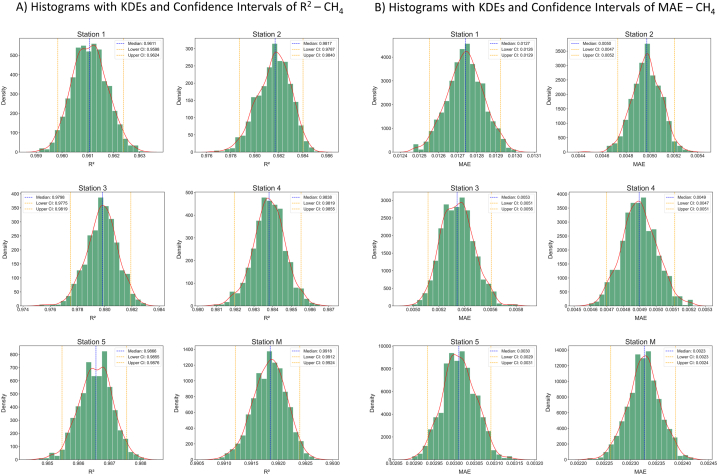
A) R^2^ across 1,000 bootstrap samples of the training models for CH_4_, showing median and 95 % confidence intervals. B) MAE across 1,000 bootstrap samples of the training models for CH_4_, showing median and 95 % confidence intervals.

Following the calibration models for each station, the goodness of fit between the models' output concentrations and the reference instrument concentrations during the training phases (i.e., on the 70 % of datasets randomly selected to build the models) was assessed. Through the bootstrap statistical technique, the median value of the R^2^ coefficient and the MAE of each model were paired with the relative 95 % confidence intervals (reported between the square brackets [] in the following text) which allowed to evaluate the variability of the scores, and thus to assess the accuracy of the models and the uncertainty associated to their previsions. The distribution plots of both the R^2^ coefficient (Fig. 3) and the MAE (Fig. 4) were obtained through frequency histograms to which the kernel density estimates (KDE) were superimposed, using 20 classes for the histograms and the default parameters *bw_method=’scott’* and *bw_adjust =1* to calculate the bandwidth in KDE (*seaborn* library).

All the models for CO_2_ and CH_4_ calibrations were shown to be well correlated to the reference values during the training phase, with R^2^ values ranging from 0.9855 [0.9844, 0.9865] (station 5; Fig. 3A) to 0.9974 [0.9972, 0.9975] (station 1; Fig. 3A), for CO_2_ (R^2^ scores and confidence intervals for each station are reported in Fig. 3A), and from 0.9611 [0.9598, 0.9624] (station 1; Fig. 4A) to 0.9918 [0.9912,0.9924] (station M; Fig. 4A), for CH_4_ (R^2^ values and confidence intervals for each station are reported in Fig. 4A). The R^2^ distribution curves, both for CO_2_ and CH_4_ models, displayed normal distributions with narrow intervals of confidence around the median values (red dashed lines in Fig. 3, Fig. 4), demonstrating the predictive models to be robust and accurate. As just depicted, CO_2_ calibration models showed on average slightly higher values of R^2^ than those obtained for CH_4_, this may be due to the generally larger size of the datasets used to train the models, and the wider range of concentrations experienced during the training window, whilst CH_4_ values were around those of the atmospheric background. For this reason, each CO_2_ model was trained on a more diversified dataset, resulting in higher R^2^ values and a more accurate predictive ability. CH_4_ models, on the other hand, having trained on smaller and less variable datasets, produced lower, but anyway optimal, R^2^ values.

Further evaluation of calibration models' accuracy was carried out based on the MAE and its 95 % confidence interval, for both CO_2_ and CH_4_, (Fig. 3, Fig. 4B, respectively). In particular, the MAE ranged from 0.71 [0.69, 0.73] to 1.44 [1.42, 1.47] ppm for CO_2_ (stations 4 and 1, respectively; Fig. 3B) and from 0.0023 [0.00225, 0.00240] to 0.0127 ppm [0.0126, 0.0129] for CH_4_ (stations M and 1, respectively; Fig. 4B). Although the model of CO_2_ relative to station 1 showed the best R^2^ score, the MAE was higher than the other models. This may be due to training performed with a dataset that had on average higher reference concentration values (as reported in Table 1, Section 3.1). Analogously, station 1's model for CH_4_ calibration has suffered the highest MAE, but in this case, it was associated with the worst R^2^ value. Anyway, the magnitude of these MAEs can be considered more than satisfying, confirming the good performance of the calibration models thus trained. In a similar fashion to the R^2^ distributions, the MAE distributions (Fig. 4) suggested that the training models can be seen, in the first analysis, as reliable and robust.

### 4.2 Evaluation of models using test data

To test the performance of the calibration models, they were applied to the testing data that were not used for model ﬁtting (i.e., the remaining 15 % of the total dataset). This was a key step to further assess the quality and the generalization ability of the models when predicting new data, providing an unbiased sense of model effectiveness.

**Fig. 5:**
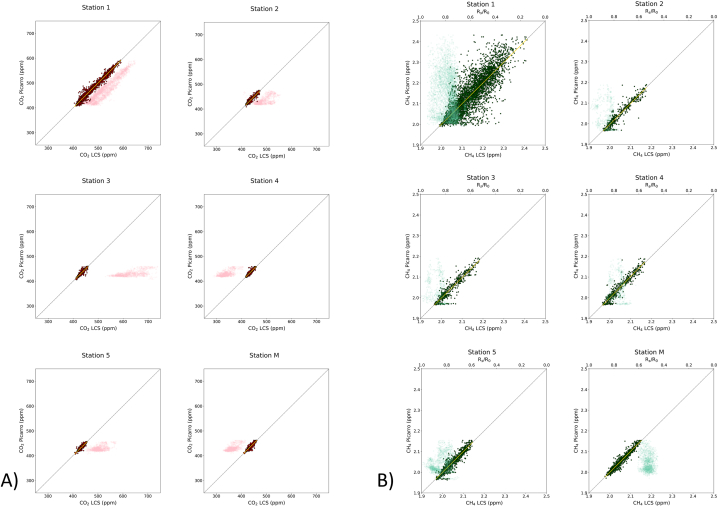
Binary plots comparing CO_2_ (A) and CH_4_ (B) concentrations resulting from correction using the calibration models (x-axis) and the actual measured reference concentrations (y-axis). Red (A) and green (B) shaded points represent, respectively, CO_2_ (in ppm) and CH_4_ (R_s_/R_0_ ratio, as pure numbers on the secondary x-axis) sensors' response before the calibration. The yellow lines represent the linear regression fit performed on the data, whilst the black dotted line is reported as the 1:1 line. The relative R^2^, MAE, and MAPE values are reported in Table 3. (For interpretation of the references to colour in this figure legend, the reader is referred to the Web version of this article.)

The binary plots in Fig. 5A and B depict, respectively, CO_2_ and CH_4_ concentrations resulting from correction using the calibration models (full points), juxtaposed with the raw sensors' signals (shaded points), alongside the actual measured reference concentrations. It is evident that linear regression fit models between the raw sensors’ signals and the reference data are entirely unsuitable, as indicated by R^2^ scores ranging from −21.3543 (station 4, Table 3) to −0.1899 (station 1, Table 3) for CO_2_ sensors, and from −2,506 (station M, Table 3) to −403 (station 5, Table 3) for CH_4_ sensors (note that the R^2^ values were computed using the function sklearn. metrics.r2_score, which can return negative values). On the other hand, the R^2^ values performed on the test data relative to CO_2_ showed excellent performances, with values ranging from 0.8781 (station 5, Table 3) to 0.9827 (station 1, Table 3), and MAE values of 2.22 and 3.76 ppm, respectively (Table 3). Regression lines integrating the test data (Table 3) had a slope (*m*) close to or equal to 1, demonstrating the efficiency of the calibration procedure. However, for some stations, the y-axis intersection (*b*) differed from 0. While this deviation from the origin intersection fell within the mean absolute error for stations 1 and 2 (Table 3), the y-axis intersection values for stations 4 and 5 were −10.03 and + 6.08 ppm, respectively (Table 3). Therefore, these shifts from zero are to be taken into account when using stations 4 and 5 for CO_2_ measurements.

**Table 3:** R^2^ scores reached by each calibration model, for both CO_2_ and CH_4_, and the relative mean absolute error (MAE) and mean absolute percentage error (MAPE). R^2^ values between the raw sensors’ response and the reference values are also reported (*R*^*2*^*raw concentrations*).

ID	CO₂					CH₄				
	R^2^ raw concentrations	R^2^ calibration	MAE	MAPE	regression line *(y = mx + b)*	R^2^ raw concentrations	R^2^ calibration	MAE	MAPE	regression line *(y = mx + b)*
Station 1	−0.1899	0.9827	3.76	0.81 %	*y = 1.00x - 0.85*	−471	0.7312	0.034	1.58 %	*y = 1.00x - 0.000*
Station 2	−4.0782	0.9467	2.19	0.50 %	*y = 1.01x - 2.36*	−603	0.8988	0.012	0.60 %	*y = 1.01x - 0.025*
Station 3	−20.3856	0.8906	2.24	0.52 %	*y = 0.99x + 3.58*	−553	0.8830	0.013	0.66 %	*y = 1.00x + 0.006*
Station 4	−21.3543	0.9167	1.95	0.45 %	*y = 1.02x - 10.03*	−804	0.9077	0.012	0.60 %	*y = 1.03x - 0.061*
Station 5	−13.1229	0.8781	2.22	0.52 %	*y = 0.99x + 6.08*	−403	0.9016	0.008	0.40 %	*y = 1.01x - 0.012*
Station M	−15.4463	0.8969	2.14	0.50 %	*y = 1.01x - 4.05*	−2507	0.9410	0.006	0.30 %	*y = 1.01x - 0.030*

Compared to CO_2_, the calibrations on CH_4_ data achieved a lower correlation, with data predictions relatively more dispersed than the reference values (R^2^ values ranging from 0.7312 to 0.9410, and corresponding MAEs of 0.03 and 0.01 ppm, for station 1 and M respectively; Table 3), but slope and y-axis intersection values close to 1 and 0 ppm, respectively (Table 3). As aforementioned, this limitation stems from the relatively smaller training datasets gather for CH_4_ and the reduced variability in sensor-recorded concentrations. Moreover, although station 1 had the highest counts in the CH_4_ datasets, it exhibited the poorest performance during the test phase, reflecting the lowest scores achieved in the training window (Section 3.1). This could potentially be improved through further hyperparameter tuning, which may not have yet yielded the optimal results, and taking into account other potential interferents not considered in this study. Nevertheless, the results are highly promising, yielding the model's best generalization to date. This enables us to detect concentration fluctuations at levels as low as tens of ppb, even against a backdrop of background CH_4_ values, a level of sensitivity and precision that would not have been expected based on the premises of the sensor manufacturer's datasheet.

**Fig. 6:**
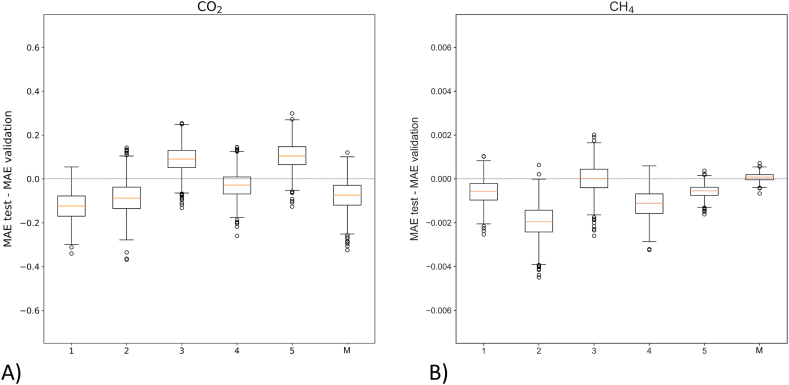
Boxplots reporting the difference between the MAE calculated from the test data corrected with the calibration models and the MAE calculated, across 1000 bootstrap samples, on the validation data, for CO_2_ (A) and CH_4_ (B), respectively. A value of MAE test – MAE validation close to zero (dashed line) points out a low degree of overfitting.

Moreover, the MAEs calculated on the test and validation datasets have been compared during the post-training phase (Fig. 6), to evaluate if the models are not subject to overfitting. The MAEs calculated on the validation and test dataset are comparable, in fact, the differences in *MAE test – MAE validation* are in a small range around 0, which points out a low degree of overfitting [59].

## 5 Conclusions

In recent years, increasing awareness of the harmful impact of air pollution on human health, the global climate, and ecosystems has emphasized the need to seek cost-effective approaches for measuring and monitoring air pollution, able to increase the availability of high-density and comprehensive data across time and space. This study demonstrates that the LFR machine learning algorithm, when applied to low-cost CO_2_ and CH_4_ sensors, can provide accurate data to evaluate air quality. Table 4 displays the performance of the LFR calibration, determined in this study, along with results from other calibration studies that used sensors with the same operating principles [15,17,23,60,61]. It is noteworthy that studies involving machine learning show, on average, the highest R^2^ scores, pinpointing that non-parametric regression models are better suited to address the challenges imposed by low-cost sensors. The approach proposed for the quantification of CO_2_ and CH_4_ in this study showed marked improvement relative to previous efforts, with models' output exhibiting excellent correlations with the reference values (R^2^ values exceeding 0.8781 for CO_2_ and 0.7312 for CH_4_, respectively). Such high correlation coefficients underline the model's effectiveness in capturing variations in atmospheric gas concentrations. Furthermore, the fractional error of the proposed models at a 1-min time resolution was minimal, with less than 1 % for CO_2_ and between 0.3 % and 2 % for CH_4_. These small fractional errors corresponded to mean absolute errors of less than 4 ppm for CO_2_ and less than 40 ppb for CH_4_. This analytical precision is fundamental for air quality monitoring and understanding the evolution of greenhouse gases, whose even minor fluctuations in concentration levels can have significant implications. Very good results were achieved also by Ref. [23] (Table 4) through a two-step calibration approach, involving several linear, power, and Michaelis Menten-based equations (Table 4, mean R^2^ values between 0.58 and 1.00). However, it's important to note that their calibration setup was conducted under laboratory conditions. Furthermore, their study focused on using the Figaro TGS 2611-E00 for measurements in flux chambers, and the equations they propose may not be optimized for CH_4_ background concentrations [23].

**Table 4:** Performance (R^2^ values) of regression models on test data from this study and previous studies [15,17,23,60,61] using CO_2_ and CH_4_ low-cost sensors.

Target gas	Sensor	Study	Regression type	R^2^	Study location
CO₂	ELT S–100H	Spinelle et al., 2017	LR	0.021-0.71	Po Valley, Italy
			MLR	0.16	
			ANN (machine learning)	0.79	
	ELT S-100/300	Casey et al., 2019	ANN (machine learning)	0.85	Greeley, CO
	Sensirion NDIR SCD 30	This study	LFR (machine learning)	0.73-0.94	Several locations in Italy
CH₄	Figaro TGS 2600	Eugster and Kling, 2012	LM	0.2	Toolik Lake, AK
	Figaro TGS 2600	Collier-Oxandale et al., 2018	Inverted LM	0.37-0.76	Los Angeles, CA
	Figaro TGS 2600	Collier-Oxandale et al., 2018	Inverted LM	0.33-0.46	Platteville, CO
	Figaro TGS 2600	Casey et al., 2019	ANN (machine learning)	0.66	Greeley, CO
	TGS 2611-E00	Bastviken et al., 2020	Step 1: linear, power and Michaelis-Mented equations	0.58-1.00	Laboratory experiments (flux chambers)
			Step 2: linear and power functions		
	TGS 2611-E00	This study	LFR (machine learning)	0.88-0.98	Several locations in Italy

The successful application of the LFR model to CO_2_ and CH_4_ low-cost sensor data indicates the potential of this approach for widespread use in air quality monitoring, both in research and practical applications. In fact, the accuracy and cost-effectiveness of this method make it a valuable tool for identifying trends and mitigating air pollution in various settings, possibly integrating the monitoring stations with sensors for other air contaminants (e.g., PM, NO_x_, CO, etc.). However, there are still avenues for further improvement and exploration in this field. The study findings suggest that additional tuning of hyperparameters could enhance the performance of some models, potentially reducing the fractional error even further. Moreover, although the proposed calibration approach offers promising results with relatively straightforward implementation, site-specific data collection would be necessary to strengthen the calibration dataset before employing these stations and calibration for studying or monitoring purposes. Additionally, a key improvement will involve integrating the low-cost stations with remote data transmission modules LoRaWAN type, a low-energy communication protocol based on radio waves that will enable the seamless uploading of air quality data onto a centralized web server, facilitating real-time access, control, and processing for end-users. This perspective is pivotal to ensure a network of monitoring low-cost stations capable of overcoming the spatial heterogeneity that afflicts the current monitoring systems. Indeed, the empowerment with real-time accessibility to comprehensive air quality data would be instrumental in several domains. Firstly, it could assist regulatory bodies and policymakers in monitoring and implementing environmental standards. Secondly, it could equip researchers with updated and high-resolution data to manage pollutant species studies and forecasting models. Finally, it could provide a strong foundation for didactic purposes, enabling communities to actively engage in environmental awareness and prompt actions to safeguard public health.

## Data availability statement

Data are included in the article's Supplementary Material section.

## CRediT authorship contribution statement

**R. Biagi:** Writing – original draft, Visualization, Validation, Software, Methodology, Investigation, Formal analysis, Data curation, Conceptualization. **M. Ferrari:** Writing – original draft, Validation, Software, Methodology, Formal analysis, Data curation, Conceptualization, Investigation. **S. Venturi:** Writing – review & editing, Validation, Methodology, Data curation, Conceptualization, Investigation. **M. Sacco:** Conceptualization, Methodology, Software, Writing – review & editing. **G. Montegrossi:** Writing – review & editing, Methodology, Conceptualization. **F. Tassi:** Project administration, Methodology, Conceptualization, Validation, Writing – review & editing, Supervision.

## Declaration of competing interest

The authors declare that they have no known competing financial interests or personal relationships that could have appeared to influence the work reported in this paper.
