# Supplementary material for: Development and machine learning-based calibration of low-cost multiparametric stations for the measurement of CO2 and CH4 in air
Source: Heliyon. 2024 Apr 24;10(9):e29772. doi: 10.1016/j.heliyon.2024.e29772 (PMC11076643; doi:10.1016/j.heliyon.2024.e29772)
Supplement: Multimedia component 7 [file mmc7.docx]

**Technical note: circuit wiring for a CO_2_-CH_4_ low-cost station**

*Supplement of*

**Development and machine learning-based calibration of low-cost multiparametric stations for the measurement of CO_2_ and CH_4_ in air**

Biagi R.*^1*^*, Ferrari M.*^1^*, Venturi S.*^1,2,3^,* Sacco M.*^4^*, Montegrossi G.*^2^*, Tassi F.*^1,2^*

*^1^Department of Earth Sciences, University of Florence, Via G. La Pira 4, 50121 Firenze, Italy*

*^2^Institute of Geosciences and Earth Resources (IGG), National Research Council of Italy (CNR), Via G. La Pira 4, 50121 Firenze, Italy*

*^3^Istituto Nazionale di Geofisica e Vulcanologia, Sezione di Palermo, Via Ugo La Malfa 153, Palermo 90146, Italy*

*^4^Department of Physics and Astronomy, University of Florence,* *Via Sansone 1, 50019 Sesto Fiorentino, Firenze, Italy.*

* Corresponding author. Department of Earth Sciences, University of Florence, Via G. La Pira 4, 50121 Firenze (Italy). Tel: +39 3316312879. E-mail: rebecca.biagi@unifi.it

Submitted to Heliyon

**Read first**

This description serves as an illustrative example intended to inspire and support broader applications in greenhouse gas measurements. While the methods outlined have proven effective for us, slight disparities in electronic components across brands or batches may necessitate adjustments. Consequently, we cannot guarantee full functionality solely based on this description. Users should anticipate investing their own development time and engaging in troubleshooting. The authors are keen to learn from any issues and enhancements, and they would greatly appreciate communication with users**.**

**Hardware**

The list of parts needed for the setup of the stations is given in Table S1, and more details about the sensors' operation are given in the Manuscript. Fig. S1 illustrates the wiring scheme to connect all the components to the Arduino UNO Rev3 board.

Table S1. List of parts needed for the setup of one low-cost station for CO2, CH4, air temperature and relative humidity, with a data logger to save data on a microSD card.

| Type | Name | Brand | Qty |
| --- | --- | --- | --- |
| Processor | Arduino UNO Rev3 | Arduino | 1 |
| Connections | Breadboard 400 pin |  | 1 |
| Sensor | SCD30 CO2/Temp/Humidity Sensor | Sensirion | 1 |
| Sensor | NGM2611-E13 | Figaro | 1 |
| Sensor | DHT22 | Adafruit | 1 |
| Data logger | MicroSD Card Breakout Board | Adafruit | 1 |
| Data logger | MicroSD 32 GB |  | 1 |
| Clock | DS3231 Precision RTC Breakout | Adafruit | 1 |
| Clock | Battery CR1220 per RTC |  | 1 |
| Connections | Resistor 10 kΩ |  | 1 |
| Connections | Jumper M/M |  | 20 |
| Connections | Jumper M/F |  | 20 |
| Case | PVC pipe fitting |  | 1 |

**
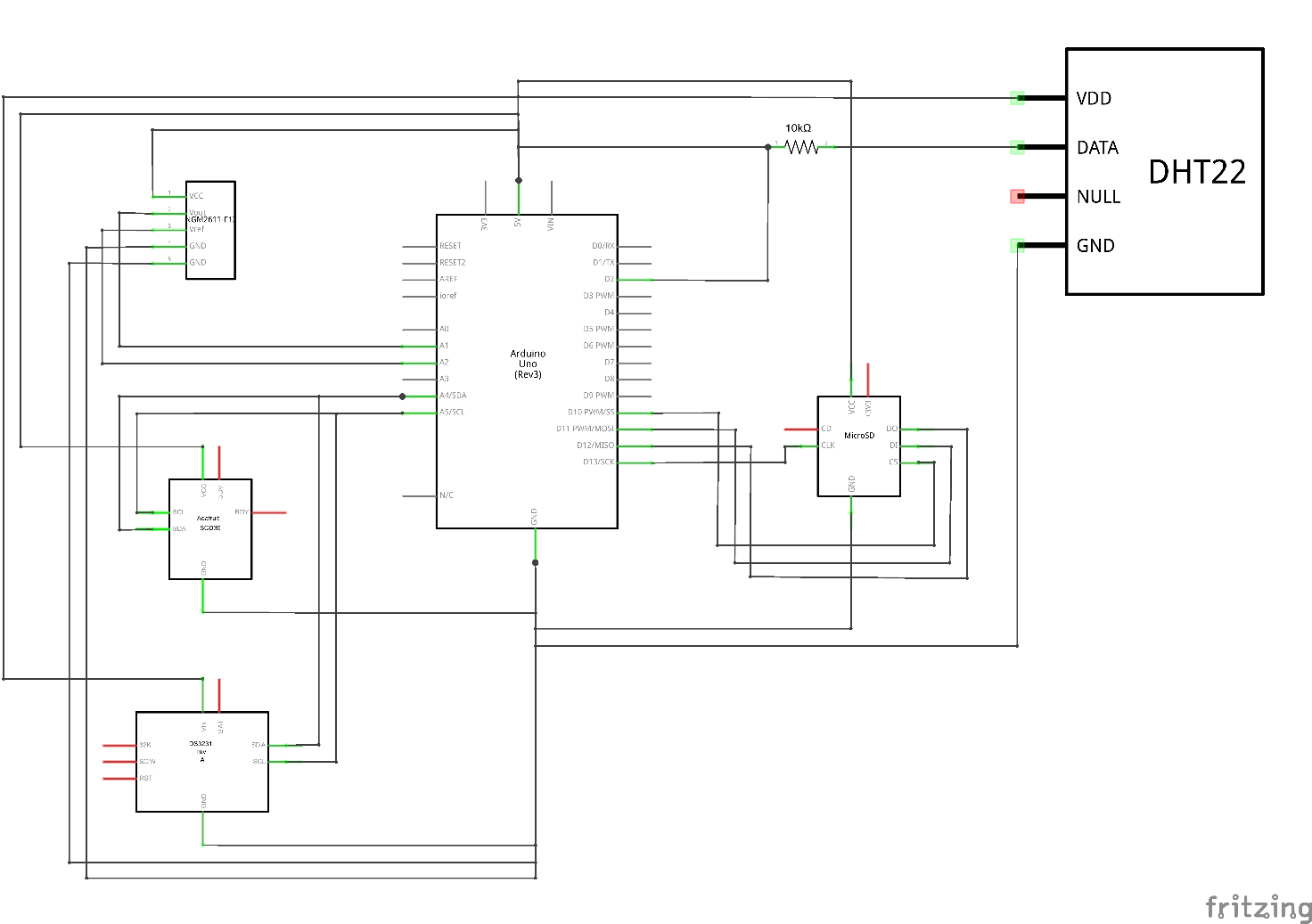
**

Figure S1. Circuit wiring for a CO_2_-CH_4_ low-cost station and environmental parameters.

**Software**

A code for Arduino was created by blending and adjusting information openly accessible from sensor manufacturers and the generous contributions of the open-source Arduino community online. You can access the code *CO2-CH4.ino* in the Supplementary Material. For guidance on fundamental Arduino software usage, numerous high-quality open-source tutorials are available on the internet.

**Power supply**

The station (Fig. S2) can be powered by a transformer cable with a voltage between 7 and 9 V. You can also power the station with an acid lead battery of 12V, taking care to add a voltage modulator to lower the battery voltage before connecting to the Arduino. The lifetime of the second configuration is about 4 weeks using a battery of 60 Ah without a solar panel. For shorter measurements, it is possible also to use a set of 9V batteries or a power bank with a USB cable. Note that with these setups, there is no error indication or alarm, when the battery level is low.

**General use**

1. Connect the components as depicted in Fig. S1.
2. Insert the microSD card into the SD card holder.
3. Install the software *Arduino IDE* by Arduino and install the required libraries.
4. Connect the station to your computer and upload the software *CO2-CH4.ino*, reported in the Supplementary Material, onto the Arduino UNO board.
   1. **At the first usage** (and in case the RTC will lose the date after a current drop), remove the comment (“//”) from line 106 and upload the code.
   2. Comment again on line 106 and reupload the code (**IMPORTANT!** This will prevent your station from starting from the same date every time the station creates a new file).
   3. Set the desired recording frequency at line 49 (milliseconds).
5. Now the station is ready to be connected to the power source that you chose.
6. The main board should now start, and LEDs should be blinking. A red LED on the data logger should blink every *n* seconds (*n* = selected interval) when data is logged to the microSD card.
7. The CO_2_ sensor LED (yellow) in the measurement cell should start blinking.
8. To turn off the device, unplug the power cable from the main Arduino board.
9. Download the data to your computer. Note that the data logger is set to create a new text file with an incremental file number every time that the station is powered, so it is not necessary to format the microSD card before using it again, avoiding the risk of overwriting if the station loses power.

**Use of the calibration notebooks**

The calibration notebooks for CO_2_ and CH_4_ are provided in the Supplementary Material section (CO2 - calibration paper.ipynb, and CH4 - calibration paper.ipynb, respectively). The codes are written in Python, and users can execute them by installing the Anaconda Navigator software and launching the Jupyter Notebook. Disclaimer: Users must update the directory path in the fourth cell according to their specific directory!


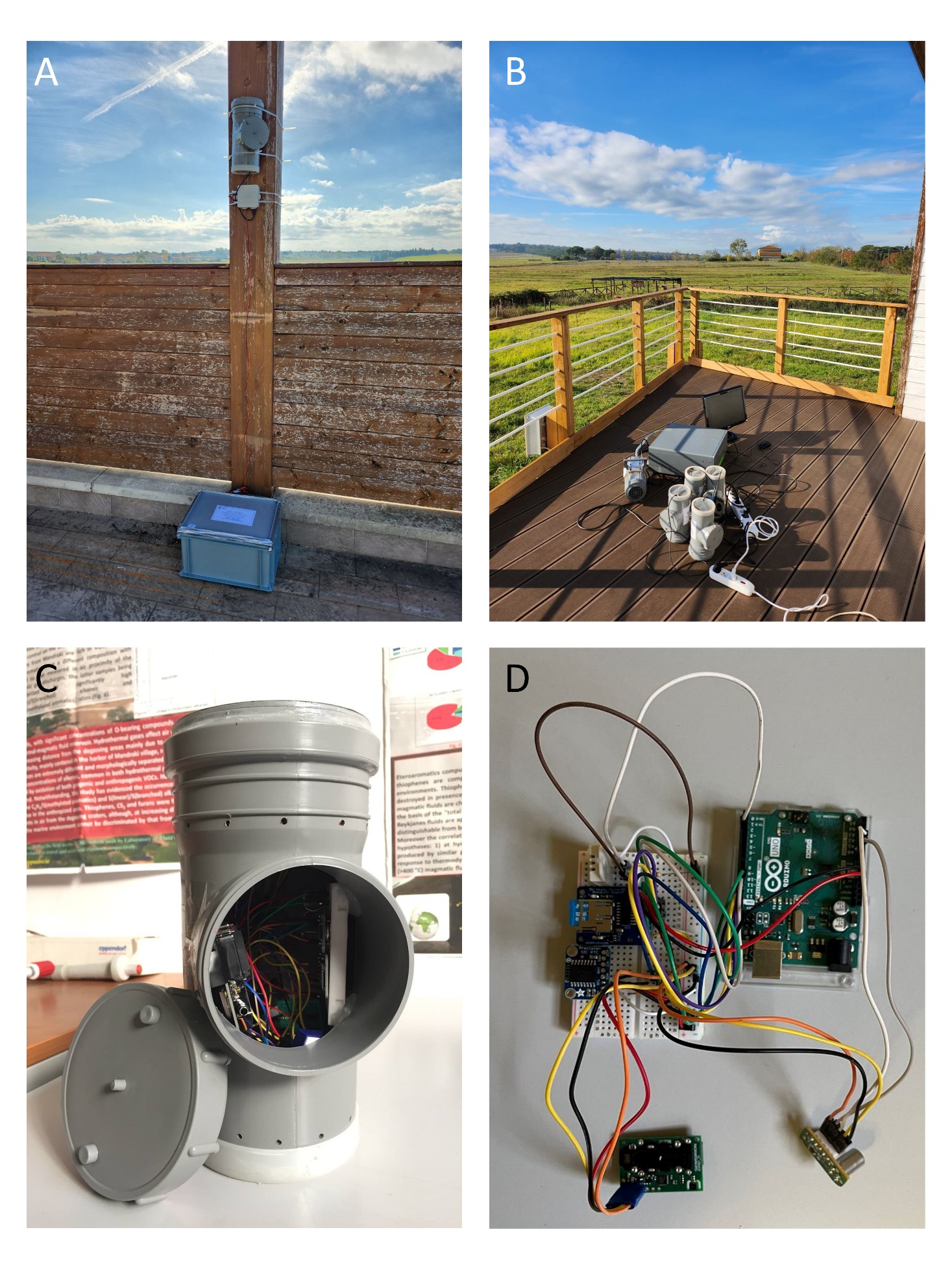


Figure S2. Photos of A) the station in operation using a 12V lead battery for the power supply, B) the acquisition of calibration datasets, C) the low-cost station’s case, and D) the circuit configuration.
